# Epidemiology and Clinical Features of Adult Patients with Psoriasis in Malaysia: 10-Year Review from the Malaysian Psoriasis Registry (2007–2016)

**DOI:** 10.1155/2018/4371471

**Published:** 2018-04-23

**Authors:** Azura Mohd Affandi, Iman Khan, Nooraishah Ngah Saaya

**Affiliations:** Department of Dermatology, Hospital Kuala Lumpur, Kuala Lumpur, Malaysia

## Abstract

**Background:**

Psoriasis is a chronic inflammatory skin disease affecting 2-3% of the general population.

**Aim:**

To evaluate the epidemiology and clinical characteristics of patients with psoriasis who seek treatment in outpatient dermatology clinics throughout hospitals in Malaysia.

**Materials and Methods:**

Data were obtained from the Malaysian Psoriasis Registry (MPR). All patients (aged 18 and above) who were notified to the registry from July 2017 to December 2017 were included in this study.

**Results:**

Among 15,794 patients, Malays were the most common (50.4%), followed by Chinese (21.4%), Indian (17.6%), and others (10.6%). The mean age onset of psoriasis for our study population was 35.14 ± 16.16 years. Male to female ratio was 1.3 : 1. 23.1% of patients had positive family history of psoriasis. The most common clinical presentation was chronic plaque psoriasis (85.1%), followed by guttate psoriasis (2.9%), erythrodermic psoriasis (1.7%), and pustular psoriasis (1.0%). Majority of our patients (76.6%) had a mild disease with BSA < 10%. 57.1% of patients had nail involvement, while arthropathy was seen in 13.7% of patients. Common triggers of the disease include stress (48.3%), sunlight (24.9%), and infection (9.1%). Comorbidities observed include obesity (24.3%), hypertension (25.6%), hyperlipidemia (18%), diabetes mellitus (17.2%), ischaemic heart disease (5.4%), and cerebrovascular disease (1.6%). The mean DLQI (Dermatology Life Quality Index) was 8.5 ± 6.6. One-third (33.1%) of the patients had a DLQI score of more than 10, while 14.2% of patients reported no effect at all.

**Conclusion:**

Our study on the epidemiological data of adult patients with psoriasis in Malaysia showed a similar clinical profile and outcome when compared to international published studies on the epidemiology of psoriasis.

## 1. Introduction

Psoriasis is a chronic, immune-mediated inflammatory skin disease that is often associated with systemic manifestations. It is a lifelong disease that can have negative impact on patients' quality of life. Psoriasis has a strong genetic component but environmental factors play an important role in the presentation of this disease [1, 2].

Psoriasis affects approximately 2.0% to 3.0% of the world's population [3–5]. To date, epidemiological studies have demonstrated variable prevalence among different population and ethnic groups worldwide. Higher prevalence rates were found in western countries; the distribution ranges from 2.2% in the U.K [6] to as high as 4.5% in Norway [7]. The prevalence of psoriasis among patients in the United States was 2.2% to 3.15% [8], while lower rates were observed in Latin Americans, Indians, Africans (Egypt and Tanzania) [9], and in Asia at less than 0.5% [10–12]. The wide variation in estimates of prevalence between regions may be attributed to the differences in ethnic or racial composition, genetics, and environmental and climate conditions [13, 14].

Although the number of studies conducted locally is increasing, there is still limited information concerning the epidemiological and clinical data pertaining to psoriasis. Informed data prevalence may contribute to a better understanding of the disease burden, updating population research, and advancement of health policies. Thus, this study aims to define the epidemiology, clinical profile, and the impact on quality of life among adult patients with psoriasis in Malaysia.

## 2. Materials and Methods

This was a multicenter study involving adult patients with psoriasis (aged 18 and above) attending 25 dermatology outpatient clinics throughout Malaysia from July 2007 to December 2016. Data collection was based on the Malaysian Psoriasis Registry (MPR). The MPR is a prospective, ongoing, systematic collection of data on patients with psoriasis in Malaysia. Data was collected at baseline and every 6 months. However, follow-up data was not available for all patients, as some were notified only once. The diagnosis of psoriasis is made based on clinical evaluation. Confirmation of diagnosis by histopathology examination is optional. Data were analyzed using descriptive analyses for sociodemographic characteristics of the patients, aggravating factors, comorbidities, types of psoriasis, and treatment modalities.

The disease severity was assessed using the body surface area (BSA) involvement and presence of nail and joint involvement. The impact of skin symptoms on quality of patient's life was evaluated using the Dermatology Life Quality Index (DLQI). The score ranges from 0 to 30 and severe impact on health-related quality of life (HRQoL) by psoriasis is defined as a DLQI score ≥ 10 [15].

### 2.1. Statistical Analysis

Descriptive statistics were presented as number and percentages for categorical variables. Mean with standard deviation (SD) was used for normally distributed data, while median with interquartile range (IQR) was used for data that were not normally distributed. Collected data was tabulated using SPSS.

## 3. Results

There were a total of 15,794 patients (aged 18 years and above) notified to the MPR between July 2007 and December 2016. 56.6% of the patients were male and 43.4% were female patients. The racial distributions of patients were Malay (50.5%), Chinese (21.4%), Indian (17.6%), and others (10.6%) (Table 1).

Psoriasis can present at any time of life. Female patients had an earlier age of onset of psoriasis, with a mean age of 32.59 ± 16.64, compared to male, with a mean age of 37.09 ± 15.51. Positive family history was reported in 23.1% of patients, and female patients had a higher percentage of family members with psoriasis, compared to male patients (Table 1).

**Figure 1:**
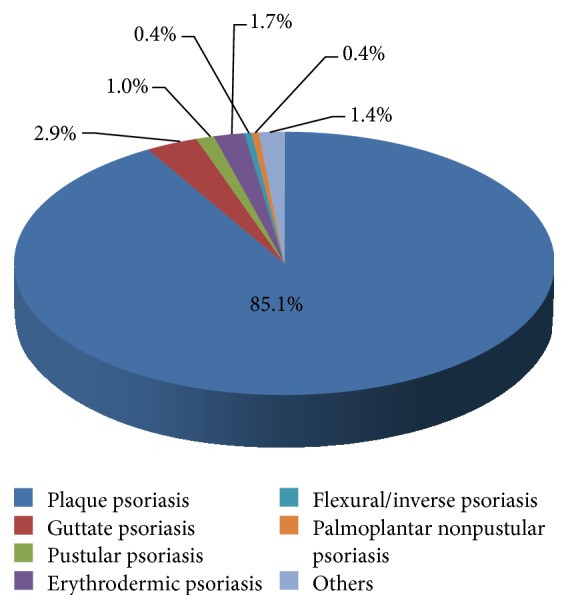
Type of psoriasis.

Psoriasis can have several presentations. The most common type of psoriasis in our study was plaque psoriasis (85.1%), followed by guttate psoriasis (2.9%), erythrodermic psoriasis (1.7%), and pustular psoriasis (1.0%). Other less common types include flexural psoriasis and palmoplantar nonpustular psoriasis, each with 0.4% (Figure 1).

In terms of disease severity, 25.2% of patients had a body surface area (BSA) of less than 5%, while 51.4% of our patients had BSA involvement of 5–10%, and 21.7% of patients had a BSA of >10–90%. Only 1.8% were reported to be erythrodermic (BSA > 90%). Male patients had more severe disease (BSA > 10%), compared to female patients (26.7% and 19.6%, resp.) (Table 1).

Of 15,794 patients, 57.1% of patients had nail involvement, and the percentage is higher in male patients, compared to female ones (62.0% and 50.6%, resp.). Nail pitting was the most common form (72.3%) followed by onycholysis (48.3%), nail discolouration (29.4%), subungual hyperkeratosis (12.6%), and total nail dystrophy (4.8%) (Table 1).

Psoriatic arthropathy was reported in 2,168 (13.7%) patients. Psoriatic arthropathy was more commonly reported in female compared to male patients (16.3% and 11.8%, resp.).

Oligo/monoarthropathy (37.9%) was the most common type, followed by symmetrical polyarthropathy (30.6%), distal hand joint arthropathy (29.6%), spondylitis/sacroiliitis (7.4%), and arthritis mutilans (2.8%) (Table 1).

52% of patients in our study had identifiable aggravating factors. Most of the patients confirmed stress as an aggravating factor (48.3%), followed by sunlight (24.9%) and infection (9.1%). Other less common aggravating factors include smoking, trauma, drugs, alcohol, and pregnancy.

3,377 (24.3%) of our patients were obese (BMI >= 30). Other common comorbidities associated with psoriasis in patients in this study were hypertension (25.6%), hyperlipidemia (18%), diabetes mellitus (17.2%), ischaemic heart disease (5.4%), and cerebrovascular disease (1.6%) (Table 1).

## 4. Treatment

Data on types of therapy was available for 15,635 patients at baseline/first visit. However, only 5,701 patients have complete data on types of therapy at last follow-up (6 months and above). Between 93.6% and 95.4% were prescribed topical treatment at baseline and at follow-up. There were not many differences in the types of topical treatment prescribed at baseline and at follow-up. Topical steroid was the most common treatment prescribed, followed by emollients and tar preparation. Other types of topical treatments include keratolytics, calcipotriol, calcipotriol with betamethasone dipropionate, and dithranol (Table 2).

A total of 449 (2.9%) patients received phototherapy at baseline and the number reduced to 1.2% at last follow-up. Since phototherapy was given for 3-4 months only, this could be an explanation as to why the number of patients receiving phototherapy during last visit was less, since notification to the registry was made at 6 months and above. Most of the patients received Narrow Band UVB (NBUVB). Not many patients had oral/topical/bath PUVA (Table 2).

Systemic therapy was required in 2,910 (18.5%) patients at baseline. During last follow-up (6 months and above), the percentage of patients on systemic therapy reduced to 8.9% only. This might be explained by missing data, as data for follow-up was only available for 5,701 patients. The most common systemic agents given were methotrexate, followed by acitretin, sulphasalazine, and cyclosporine. Biologics therapy was given in 3.3% of patients at baseline and 2.4% of patients continued the treatment at last follow-up (6 months and above). The three most common biologics given were ustekinumab, adalimumab, and etanercept (Table 2).

Despite the treatments instituted, there was not much difference in the outcomes of the patients as measured by BSA affected during follow-up (6 months and above). However, the amount of data available during follow-up was a lot less than baseline (15,635 patients at baseline and 5,701 patients during last follow-up). Even though there was not much difference in terms of BSA affected, there was a reduction in mean DLQI at baseline and follow-up (8.51 ± 6.58 and 8.29 ± 6.56, resp.) (Table 2).

## 5. Quality of Life

**Figure 2:**
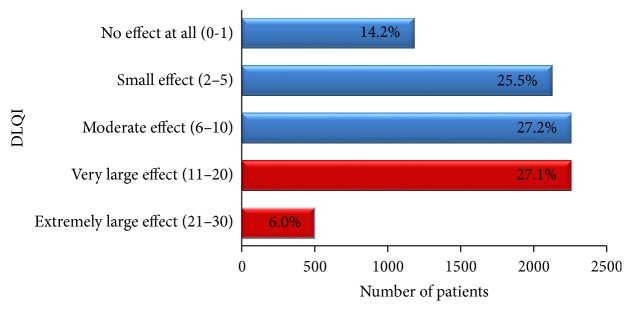
Quality of life in adult patients with psoriasis.

Psoriasis can impose major psychological impact on patients and affect their quality of life. The mean DLQI for our patients was 8.5 ± 6.6. There was not much difference in mean DLQI between males and females (Table 1). One-third (33.1%) of patients scored more than 10, which indicates severe impairment of quality of life, while 14.2% of patients reported no effect at all. Patients who reported small effect and moderate effect were 25.2% and 27.2%, respectively (Figure 2).

## 6. Discussion

### 6.1. Demography

Psoriasis occurs worldwide, affecting men and women of all ages and ethnic origins. It can present at any age and usually persists for life. Several large studies have found that the age of onset for psoriasis has a bimodal distribution (early and late onset) [16–18]. It is suggested that the bimodal distribution of psoriasis incidence represents two clinical presentations of the disease, type I (early onset) presenting at <40 years of age and type II (late onset) at >40 years of age [16]. This bimodality, however, was not observed in our study. The mean age of onset of psoriasis in our population was 35.14 ± 16.16, which is consistent with other studies that reported that psoriasis generally appears in the third decade [19, 20]. The male to female ratio in our study revealed a slightly higher incidence in men than in women at 1.3 : 1, which was similar to a study by Ejaz et al. [19] who reported a male to female ratio of 1.2 : 1. Although most studies that reported prevalence by sex showed no significant difference in the frequency of psoriasis between genders [9, 11, 12, 21, 22], there are some studies that showed a higher incidence in women than in men [9].

### 6.2. Clinical Features

Plaque psoriasis was the most common type of psoriasis, accounting for 85.1% of patients, followed by guttate psoriasis (2.9%), erythrodermic psoriasis (1.7%), and pustular psoriasis (1.0%).

Nail involvement is common in patients with psoriasis. The prevalence of psoriasis-related nail disease was observed in 57.1% of our patients. The most common nail changes seen were pitting, seen in 72.3% of our patients, followed by onycholysis (48.3%), nail discolouration (29.4%), subungual hyperkeratosis (12.6%), and total nail dystrophy (4.8%). In contrast, a cross-sectional study by Brazzelli et al. [23] in Italy reported onycholysis as the most common nail change. However, the exact prevalence of specific nail patterns in psoriatic patients is still relatively underreported.

### 6.3. Obesity and Psoriasis

In the present study, we found 24.3% of psoriasis patients with obesity. Numerous studies have implicated a close association between obesity and psoriasis. In a systematic review and meta-analysis by Armstrong et al. [24], it was reported that psoriasis patients have >50% likelihood for obesity compared to those without psoriasis. Similarly, in a recent study, Helmick et al. [25] found that the prevalence of obesity in patients with psoriasis was also reported to be higher than the general population.

So far, the role of weight loss as treatment for psoriasis in obese patients is unclear; however, it is reasonable to assume that weight loss in such patients may reduce the obesity-induced inflammation, which may in turn improve the skin disease [26].

### 6.4. Comorbidities

Epidemiological studies revealed that patients with psoriasis have an increased risk of developing comorbidities related to the metabolic syndrome which include arterial hypertension and abnormalities in lipid and glucose metabolism [27].

In this study, our patients were shown to have hypertension (25.6%), hyperlipidemia (18%), diabetes mellitus (17.2%), ischaemic heart disease (5.4%), and cerebrovascular disease (1.6%). Similar findings were also reported in a study by Cohen et al. [27], which involved 16,851 patients with psoriasis and 48,681 controls. The study reported hypertension in 27.5% of the patients with psoriasis and 14.4% of the controls (*p* < 0.001), while diabetes mellitus occurred in 13.8% of psoriasis patients as compared to 7.3% of the controls (*p* < 0.001). In a cross-sectional study by Shapiro et al. [28] on 46,095 patients with psoriasis (case patients) and 1,579,037 subjects without psoriasis (control patients), it was revealed that diabetes was significantly higher in psoriasis patients as compared with the control group (odds ratio [OR]: 1.27, 95% confidence interval [CI]: 1.1–1.48). Atherosclerosis was also found to be significantly higher in psoriasis patients as compared to the control group (OR: 1.28, 95% CI: 1.04–1.59).

Numerous studies have shown a great correlation between the prevalence of metabolic syndrome and individuals with psoriasis. Given the serious complications, screening for metabolic syndrome should be included in the long-term management of individuals with psoriasis.

### 6.5. Stress and Psoriasis

Up to 48.3% patients in our study described stress as the main triggering factor for their disease. Psoriasis exacerbations have been frequently related to acute stressful events. The normal physiological response to stress involves activation of the hypothalamus-pituitary-adrenal (HPA) axis and sympathetic adrenomedullary (SAM) axis, both of which interact with immune functions [29]. In normal individuals, stress usually elevates stress hormones (i.e., raised cortisol levels). However, there are available studies to suggest that HPA responses are reduced, while SAM responses are upregulated in psoriasis patients exposed to stress [29–31]. A study by Evers et al. [32] found that psoriasis patients had significantly lower cortisol levels at moments when daily stressors are at peak levels. The study also reported that psoriasis patients with high levels of daily stressors exhibited lower mean cortisol levels, as compared to psoriasis patients with low levels of daily stressors [32].

Other exacerbating factors include sunlight (24.9%) and infection (9.1%). Smoking, trauma, drugs, alcohol, and pregnancy were reportedly less common. Although our study did not show any strong relationship of smoking with psoriasis, there are several studies that have shown a significant association of smoking being a predisposing environmental factor for the development of psoriasis [3]. The severity of psoriasis seems to be correlated with the intensity of smoking [12].

### 6.6. DLQI and Psoriasis

The Dermatology Life Quality Index (DLQI), despite methodological limitations, is currently the most commonly used method for evaluating quality of life for patients with skin conditions. The term quality of life, or health-related quality of life (HRQoL), refers to a quantitative estimation of the global impact of a disease on physical, social, and psychological well-being of a patient [3]. Factors such as disease severity, gender, age, anatomical sites of lesion, presence of comorbidities, and psychological distress can all be associated with reduced HRQoL in people with psoriasis [33].

Patients in our study had a mean DLQI score of 8.5 ± 6.6. One-third (33.1%) of our patients had a DLQI score of more than 10, which indicates severe impairment. This was similar to a local study on 223 patients, evaluating the HRQoL using DLQI [34]. The study reported 67 patients (30%) with severe impairment (DLQI score = 11–30) and a median DLQI score of 7. A study conducted in Germany reported marked impairment of health-related life quality, with a mean DLQI of 8.6. The study also reported 32% patients with QoL score of more than 10, while 15.2% had no effect on their QoL [35].

### 6.7. Psoriatic Arthropathy

Psoriatic arthritis (PsA) is a seronegative arthritis that is associated with psoriasis. PsA affects the quality of life of patients and has a considerable impact on annual healthcare expenditure and results in increased risk of mortality. Several researches have reported the prevalence of psoriatic arthropathy (PsA) in psoriasis patients, varying from 6.25% to 48% in western countries and from 1% to 9% in Asian countries [36, 37]. The differences in the prevalence can possibly be explained by geographical and ethnic differences and the differences in the applied diagnostic criteria of PsA [38]. Joint involvement was reported in 13.7% of our patients. Our prevalence was higher than that in reported studies from other Asian countries. However, our data was mainly based on history and clinical examination. The other studies focused more on psoriatic arthropathy and more stringent inclusion criteria were employed.

Moll and Wright first described five clinical patterns among patients with PsA: distal hand joint arthropathy, asymmetrical oligo/monoarthritis, symmetrical polyarthritis, spondylitis/sacroiliitis, and arthritis mutilans [39]. The exact frequency of the patterns is variable but oligoarthritis was the most commonly reported pattern [39]. Comparably, in our study, oligo/monoarthritis (37.3%) was the most common pattern, followed by symmetrical polyarthritis (30.6%), distal hand joint arthropathy (29.6%), spondylitis/sacroiliitis (7.4%), and arthritis mutilans (2.8%).

A very recent study by Mishra et al. [38] used four different types of screening tools to diagnose PsA and found that the most common pattern of PsA was symmetrical polyarthritis (46%) followed by oligoarthritis (44%). Regardless of the differences in prevalence of PsA, early screening for PsA is necessary for prevention of joint damage. In patients who were diagnosed with PsA early in arthritis clinics, up to 47% have been shown to develop erosions within the first 2 years [40]. However, patients treated within 2 years of diagnosis have less progression of joint damage than those treated 2 years after diagnosis [41]. Thus, early diagnosis has the potential to prevent joint damage and improve patient outcomes.

### 6.8. Treatment in Psoriasis

Treatment of psoriasis is based on controlling the symptoms. Topical therapies such as corticosteroids, vitamin D analogs, keratolytics, and tar preparation are useful for treating mild-to-moderate psoriasis. More severe psoriasis may be treated with phototherapy or may require systemic therapy.

A large majority of our patients were prescribed with topical treatments as first-line therapy, the most common being topical corticosteroid (75.3%). 2.8% of patients received phototherapy, while 18.4% of patients received systemic therapy. Our data was closely similar to other published studies. A retrospective study by Gillard and Finlay, based on data from a primary-care medical record database of psoriasis patients from 2002 to 2003 in the UK, demonstrated that only 4% of 6120 patients with psoriasis received either phototherapy or systemic therapy [42]. Majority of their patients (93.6%) received topical treatment only.

## 7. Conclusion

This study describes the sociodemographic characteristics, clinical features, and the impact of disease in patients with psoriasis in Malaysia. Our findings are similar to other published epidemiological studies on psoriasis. In the future, there is a need for more epidemiological studies, as they are an important contributor to a better understanding of the disease burden, updating population research, and advancement of health policies.

## Figures and Tables

**Table 1:** Patients' characteristics at baseline.

Variable	Male		Female		Total
	*n*	%	*n*	%	
*Number of patients*	8,947	56.6	6,847	43.4	15,794
*Ethnicity*					
Malay	4,083	45.6	3,885	56.7	7,968 (50.5%)
Chinese	2,164	24.2	1,215	17.7	3,379 (21.4%)
Indian	1,779	19.9	994	14.6	2,773 (17.6%)
Others	922	10.3	753	11.0	1,669 (10.6%)
*Mean age of onset of psoriasis*	37.09 ± 15.51		32.59 ± 16.64		35.14 ± 16.16
*Positive family history*	1,926	21.5	1,737	25.4	3,663 (23.1%)
*Types of psoriasis*					
Plaque psoriasis	7,696	86.0	5,752	84.0	13,448 (85.1%)
Guttate psoriasis	240	2.7	216	3.2	456 (2.9%)
Erythrodermic psoriasis	182	2.0	85	1.2	267 (1.7%)
Pustular psoriasis	50	0.6	105	1.5	155 (1.0%)
Flexural psoriasis	24	0.3	43	0.6	67 (0.4%)
Palmoplantar nonpustular psoriasis	36	0.4	23	0.3	59 (0.4%)
*Severity of psoriasis*					
BSA < 5%	1,029	25.9	801	24.4	1,826 (25.2%)
BSA 5–10%	1,882	47.5	1,843	56.0	3,725 (51.4%)
BSA > 10–90%	972	24.5	600	18.2	1,572 (21.7%)
BSA > 90%	86	2.2	45	1.4	131 (1.8%)
*Nail involvement*					
Yes	5,549	62.0	3,463	50.6	9,012 (57.1%)
Pitting	4,025	72.5	2,429	70.1	6,519 (72.3%)
Onycholysis	2,792	50.3	1,561	45.1	4,353 (48.3%)
Nail discolouration	1,783	32.1	870	25.1	2,653 (29.4%)
Subungual hyperkeratosis	816	14.7	316	9.1	1,132 (12.6%)
Total nail dystrophy	333	6.0	102	2.9	435 (4.8%)
No	3,091	34.5	3,135	45.8	6,226 (39.4%)
*Joint involvement*					
Yes	1,053	11.8	1,115	16.3	2,168 (13.7%)
Oligo/monoarthropathy	407	38.7	414	37.1	821 (37.9%)
Symmetrical polyarthropathy	286	27.2	378	33.9	664 (30.6%)
Distal hand joint arthropathy	301	28.6	340	30.5	641 (29.6%)
Spondylitis/sacroiliitis	89	8.5	71	6.4	160 (7.4%)
Arthritis mutilans	39	3.7	22	2.0	61 (2.8%)
No	7,573	84.6	5,503	80.4	13,076 (82.8%)
*Comorbidities*					
Obese (BMI > 30)	1,642	20.8	1,735	28.9	3,377 (24.3%)
Hypertension	2,535	28.3	1,750	25.6	4,285 (25.6%)
Hyperlipidemia	1,817	20.3	1,195	17.5	3,012 (18.0%)
Diabetes mellitus	1,713	19.1	1,179	17.2	2,892 (17.2%)
Ischaemic heart disease	698	7.8	203	3.0	901 (5.4%)
Cerebrovascular disease	192	2.1	78	1.1	270 (1.6%)

*Mean DLQI*	8.8 ± 6.6		8.2 ± 6.6		8.5 ± 6.6

**Table 2:** Types of treatment and outcome.

Type of therapy	Baseline/first visit (*n* = 15,635)		Last follow-up (6 months and above) (*n* = 5,701)	
	*n*	%	*n*	%
*Topical*	14,741	93.6	5,441	95.4
Topical steroids	13,079	89.4	5,022	92.3
Emollient	11,526	78.8	4,360	80.1
Tar preparation	10,546	72.1	3,789	69.6
Keratolytics	8,089	55.3	3,069	56.4
Calcipotriol	2,410	16.5	968	17.8
Calcipotriol with betamethasone dipropionate	2,202	15.1	818	15.0
Dithranol	242	1.7	100	1.8
Others	208	1.4	60	1.1
*Phototherapy*	449	2.9	184	1.2
NB-UVB	391	87.1	169	91.8
BB-UVB	28	6.2	7	3.8
Oral PUVA	12	2.7	4	2.2
Topical PUVA	7	1.6	2	1.1
Bath PUVA	4	0.9	3	1.6
Excimer laser	1	0.2	0	0.0
Others	14	3.1	5	2.7
*Systemic therapy*	2,910	18.5	1,405	8.9
Methotrexate	2,154	78.0	1,012	72.0
Acitretin	515	18.7	276	19.6
Sulphasalazine	176	6.4	67	4.8
Cyclosporin	120	4.3	56	4.0
Systemic corticosteroids	108	3.9	24	1.7
Hydroxyurea	18	0.7	3	0.2
*Biologics*	90	3.3	34	2.4
Ustekinumab	31	36.5	11	32.4
Adalimumab	25	29.4	8	23.5
Etanercept	14	16.5	7	20.6
Infliximab	3	3.5	2	5.9
Golimumab	4	4.7	1	2.9
Efalizumab	1	1.2	0	0.0
Secukinumab	2	2.4	0	0.0
*Severity of psoriasis*				
BSA < 5%	1,826	25.2	666	25.7
BSA 5–10%	3,725	51.4	1,284	49.6
BSA > 10–90%	1,572	21.7	598	23.1
BSA > 90%	131	1.8	43	1.7

*Mean DLQI*	8.51 ± 6.58		8.29 ± 6.56	

## References

[1] Langley R. G. B., Krueger G. G., Griffiths C. E. M. (2005). Psoriasis: epidemiology, clinical features, and quality of life. *Annals of the Rheumatic Diseases*.

[2] Lønnberg A. S., Skov L., Skytthe A., Kyvik K. O., Pedersen O. B., Thomsen S. F. (2016). Smoking and risk for psoriasis: A population-based twin study. *International Journal of Dermatology*.

[3] Hayes J., Koo J. (2010). Psoriasis: Depression, anxiety, smoking, and drinking habits. *Dermatologic Therapy*.

[4] Perera G. K., Di Meglio P., Nestle F. O. (2012). Psoriasis. *Annual Review of Pathology: Mechanisms of Disease*.

[5] Springate D. A., Parisi R., Kontopantelis E., Reeves D., Griffiths C. E. M., Ashcroft D. M. (2017). Incidence, prevalence and mortality of patients with psoriasis: a U.K. population-based cohort study. *British Journal of Dermatology*.

[6] Seminara N. M., Abuabara K., Shin D. B. (2011). Validity of the Health Improvement Network (THIN) for the study of psoriasis. *British Journal of Dermatology*.

[7] Olsen A. O., Grjibovski A., Magnus P., Tambs K., Harris J. R. (2005). Psoriasis in Norway as observed in a population-based Norwegian twin panel. *British Journal of Dermatology*.

[8] Kurd S. K., Gelfand J. M. (2009). The prevalence of previously diagnosed and undiagnosed psoriasis in US adults: results from NHANES 2003-2004. *Journal of the American Academy of Dermatology*.

[9] Parisi R., Symmons D. P. M., Griffiths C. E. M., Ashcroft D. M. (2013). Global epidemiology of psoriasis: a systematic review of incidence and prevalence. *Journal of Investigative Dermatology*.

[10] Rachakonda T. D., Schupp C. W., Armstrong A. W. (2014). Psoriasis prevalence among adults in the United States. *Journal of the American Academy of Dermatology*.

[11] Ding X., Wang T., Shen Y., Wang X., Zhou C., Tian S. (2012). Prevalence of psoriasis in China: a population-based study in six cities. *European Journal of Dermatology*.

[12] Bo K., Thoresen M., Dalgard F. (2008). Smokers report more psoriasis, but not atopic dermatitis or hand eczema: Results from a Norwegian population survey among adults. *Dermatology*.

[13] Griffiths C. E., Barker J. N. (2007). Pathogenesis and clinical features of psoriasis. *The Lancet*.

[14] Alexis A. F., Blackcloud P. (2014). Psoriasis in Skin of Color: Epidemiology, Genetics, Clinical Presentation, and Treatment Nuances. Desai SR, Alexis A. *Journal of Clinical and Aesthetic Dermatology*.

[15] Finlay A. Y., Khan G. K. (1994). Dermatology Life Quality Index (DLQI)—a simple practical measure for routine clinical use. *Clinical and Experimental Dermatology*.

[16] Henseler T., Christophers E. (1985). Psoriasis of early and late onset: characterization of two types of psoriasis vulgaris. *Journal of the American Academy of Dermatology*.

[17] Huerta C., Rivero E., García Rodríguez L. A. (2007). Incidence and risk factors for psoriasis in the general population. *Archives of Dermatology*.

[18] Icen M., Crowson C. S., McEvoy M. T., Dann F. J., Gabriel S. E., Maradit Kremers H. (2009). Trends in incidence of adult-onset psoriasis over three decades: a population-based study. *Journal of the American Academy of Dermatology*.

[19] Ejaz A., Raza N., Iftikhar N., Iftikhar A., Farooq M. (2009). Presentation of early onset psoriasis in comparison with late onset psoriasis: A clinical study from Pakistan. *Indian Journal of Dermatology, Venereology and Leprology*.

[20] Chang Y. T., Chen T. J., Liu P. C. (2009). Epidemiological study of psoriasis in the national health insurance database in Taiwan. *Acta Dermato-Venereologica*.

[21] Gelfand J. M., Gladman D. D., Mease P. J. (2005). Epidemiology of psoriatic arthritis in the population of the United States. *Journal of the American Academy of Dermatology*.

[22] Sinniah B., Saraswathy Devi P. S., Prasant B. S. (2010). Epidemiology of psoriasis in Malaysia: A hospital based study. *Medical Journal of Malaysia*.

[23] Brazzelli V., Carugno A., Alborghetti A. (2012). Prevalence, severity and clinical features of psoriasis in fingernails and toenails in adult patients: Italian experience. *Journal of the European Academy of Dermatology and Venereology*.

[24] Armstrong A. W., Harskamp C. T., Armstrong E. J. (2012). The association between psoriasis and obesity: a systematic review and meta-analysis of observational studies. *Nutrition & Diabetes*.

[25] Helmick C. G., Lee-Han H., Hirsch S. C., Baird T. L., Bartlett C. L. (2014). Prevalence of psoriasis among adults in the U.S.: 2003-2006 and 2009-2010 National Health and Nutrition Examination Surveys. *American Journal of Preventive Medicine*.

[26] Nestle F. O., Di Meglio P., Qin J.-Z., Nickoloff B. J. (2009). Skin immune sentinels in health and disease. *Nature Reviews Immunology*.

[27] Cohen A. D., Sherf M., Vidavsky L., Vardy D. A., Shapiro J., Meyerovitch J. (2008). Association between psoriasis and the metabolic syndrome: A cross-sectional study. *Dermatology*.

[28] Shapiro J., Cohen A. D., David M. (2007). The association between psoriasis, diabetes mellitus, and atherosclerosis in Israel: A case-control study. *Journal of the American Academy of Dermatology*.

[29] Buske-Kirschbaum A., Ebrecht M., Kern S., Hellhammer D. H. (2006). Endocrine stress responses in TH1-mediated chronic inflammatory skin disease (psoriasis vulgaris)—do they parallel stress-induced endocrine changes in TH2-mediated inflammatory dermatoses (atopic dermatitis)?. *Psychoneuroendocrinology*.

[30] Evers A. W. M., Lu Y., Duller P., Van Der Valk P. G. M., Kraaimaat F. W., Van De Kerkhoft P. C. M. (2005). Common burden of chronic skin diseases? Contributors to psychological distress in adults with psoriasis and atopic dermatitis. *British Journal of Dermatology*.

[31] Richards H. L., Fortune D. G., O'Sullivan T. M., Main C. J., Griffiths C. E. M. (1999). Patients with psoriasis and their compliance with medication. *Journal of the American Academy of Dermatology*.

[32] Evers A. W. M., Verhoeven E. W. M., Kraaimaat F. W. (2010). How stress gets under the skin: Cortisol and stress reactivity in psoriasis. *British Journal of Dermatology*.

[33] World Health Organization (2016). *Global Report on Psoriasis*.

[34] Nyunt W. W. T., Low W. Y., Ismail R., Sockalingam S., Min A. K. K. (2015). Determinants of health-related quality of life in psoriasis patients in Malaysia. *Asia-Pacific Journal of Public Health*.

[35] Augustin M., Reich K., Glaeske G., Schaefer I., Radtke M. (2010). Co-morbidity and age-related prevalence of psoriasis: Analysis of health insurance data in Germany. *Acta Dermato-Venereologica*.

[36] Wilson F. C., Icen M., Crowson C. S., McEvoy M. T., Gabriel S. E., Kremers H. M. (2009). Incidence and clinical predictors of psoriatic arthritis in patients with psoriasis: A population-based study. *Arthritis Care & Research*.

[37] Tam L. S., Leung Y. Y., Li E. K. (2009). Psoriatic arthritis in Asia. *Rheumatology*.

[38] Mishra S., Kancharla H., Dogra S., Sharma A. (2017). Comparison of four validated psoriatic arthritis screening tools in diagnosing psoriatic arthritis in patients with psoriasis (COMPAQ Study). *British Journal of Dermatology*.

[39] Moll J. M., Wright V. (1973). Psoriatic arthritis. *Seminars in Arthritis and Rheumatism*.

[40] Kane D., Stafford L., Bresniham B., FitzGerard O. (2003). A prospective, clinical and radiological study of early psoriatic arthritis: An early synovitis clinic experience. *Rheumatology*.

[41] Gladman D. D., Mease P. J., Strand V. (2007). Consensus on a core set of domains for psoriatic arthritis. *The Journal of Rheumatology*.

[42] Gillard S. E., Finlay A. Y. (2005). Current management of psoriasis in the United Kingdom: Patterns of prescribing and resource use in primary care. *International Journal of Clinical Practice*.

